# Seismic Applications of Downhole DAS

**DOI:** 10.3390/s21092897

**Published:** 2021-04-21

**Authors:** Ariel Lellouch, Biondo L. Biondi

**Affiliations:** Department of Geophysics, Stanford University, Stanford, CA 94025, USA; biondo@stanford.edu

**Keywords:** DAS, distributed, seismology, downhole, seismic, exploration

## Abstract

Distributed Acoustic Sensing (DAS) is gaining vast popularity in the industrial and academic sectors for a variety of studies. Its spatial and temporal resolution is ever helpful, but one of the primary benefits of DAS is the ability to install fibers in boreholes and record seismic signals in depth. With minimal operational disruption, a continuous sampling along the trajectory of the borehole is made possible. Such resolution is highly challenging to obtain with conventional downhole tools. This review article summarizes different seismic uses, passive and active, of downhole DAS. We emphasize current DAS limitations and potential ways to overcome them.

## 1. Introduction

Seismic waves are the primary proxy for studying processes in the Earth and its structure. Among others, they can be used to resolve large-scale structures as the core, mantle, and crust, and local areas such as fault zones or hydrocarbon reservoirs. These waves are also the primary tool in understanding the mechanisms that govern earthquake generation and their link to the Earth’s structure. Seismic studies can be either active, in which human-generated sources are excited, or passive, in which the naturally occurring seismic field is recorded.

Seismological studies are traditionally conducted with seismic receivers located at or close to the surface. The most prominent examples are earthquake monitoring networks [1] and seismic exploration surveys [2]. However, such studies are inherently limited by the fact the receivers are far away from the study areas, which are deep in the subsurface. In addition, seismic waves traveling for long distances will attenuate, especially in the shallow section of the subsurface, and their frequency content will be significantly diminished by the time they reach the receivers positioned at the surface. As a result, the resolution with which one can study subsurface structures and processes deteriorates with the depth of the study area [3].

The deployment of downhole receivers can, for targets at typical depths of several km, alleviate these limitations at the expense of limiting the subsurface volume that is well illuminated by the data. They have been used, among others, for high-resolution seismic property estimation [4], microseismic monitoring during hydraulic stimulation [5], and earthquake seismology [6,7]. However, downhole deployments incur substantial operational and budgetary challenges. The receivers have to withstand conditions of extreme temperature and pressure, in which electronic components might fail [8]. As a result, receivers are often deployed for limited time periods. Their deployment and coupling to the borehole can also be challenging and time-consuming, as it primarily depends on mechanical components. It is not uncommon that some receivers are unusable, even in modern acquisitions. For the time period in which receivers are deployed, the well is effectively mobilized and cannot be used for other operations. For these reasons, downhole deployments of traditional seismometers are relatively rare, often only temporary, and even when they do occur, the extent and density of the downhole array are limited.

Distributed acoustic sensing (DAS) offers an exceptional type of seismic measurement. It provides high spatial and temporal resolution and can record dense seismic data for tens of kilometers [9]. With conventional acquisition parameters, it can record unaliased seismic wavelengths as short as a few meters. If an optical fiber is installed in the well, DAS can provide continuous, dense downhole recording without interfering with any other activity in the well. This installation ensures adequate fiber coupling and thus high signal-to-noise ratio (SNR). Fibers are also significantly more resistant to temperature and pressure and can function as sensors for years and potentially decades. For example, an optical fiber deployed in 2005 in a deep borehole was used to record seismic data in 2017 successfully [10]. There have been many successful applications of DAS using existing telecommunication fibers in both land and marine environments. DAS was used, among others, to record earthquakes [11,12,13,14,15,16,17], reconstruct subsurface structures [10,18,19,20,21,22], identify fault zones [23,24], monitor traffic [25,26], analyze oceanic microseisms and tides [27], and record thunderstorms [28]. Nonetheless, dedicated downhole deployments, which are the focus of this review, have significantly better SNR and take full benefit of the sampling resolution and density that DAS provides [29,30,31] by targeting shorter seismic wavelengths. Deep sensor deployment also overcomes the complexity and the dissipative nature of the near-surface, which is detrimental to the analysis of signals recorded at or close to the surface [26].

In this study, we review various applications of downhole DAS, in which fibers deployed in wells record high-resolution seismic signals at depth. Its first and primary use has been as an imaging tool for hydrocarbon exploration. It has proven to be a reliable source of velocity model and local structural images through Vertical Seismic Profiling (VSP). DAS has also been used for passive seismic monitoring of both natural and human-made seismic activity. Finally, its deployment inside unconventional hydrocarbon reservoirs has allowed for revolutionary developments in reservoir imaging and characterization. Downhole DAS fibers can also be used for other types of measurements, such as temperature [32] and static strain [33]. These measurements are based on different optical phenomena, requiring different interrogators from DAS and outside the scope of this review paper.

**Figure 1:**
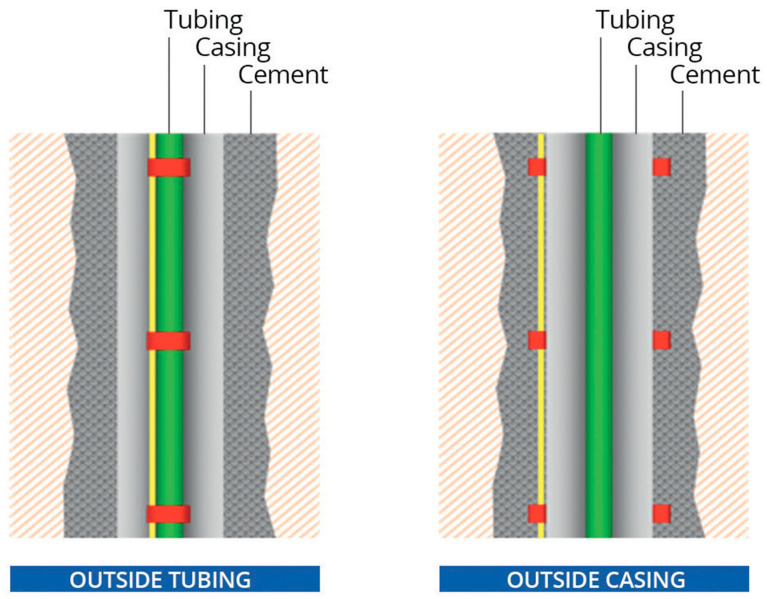
Different types of DAS installation. Elements of the well are marked, and the fiber is in yellow. Figure from Naldrett et al. (2020), First Break.

Downhole fiber deployment methods can be classified into three main groups (the first two are shown in Figure 1). The optimal deployment is outside casing (also named behind casing). As the area outside of the casing is often cemented, the coupling of the fiber to the subsurface formations is strong, and the SNR is high [34]. This method requires the installation of the fiber at an early stage. An alternative deployment, suitable for recording in existing wells, is a semipermanent fiber installation outside of the production tubing [35]. This type of deployment suffers from strong noise in the form of tubing ringing, which can be dealt with during data processing stages [36]. Nonetheless, coupling to the surrounding formations is weaker, and signal quality is lower [37]. Deployment inside the tubing is fully retrievable and less prone to failure, but noise levels are very high due to direct interaction with flowing fluid, among other factors [38,39]. The last group of deployments does not directly attach the fiber to the well. For wireline [40] deployments in vertical wells, the fiber is spiraled down the well, and coupling is obtained at the point of contact with the casing. In horizontal or deviated wells, gravitational coupling at the bottom of the well provides reliable measurements without the need for outside casing installation [41]. The same approach is useful for DAS recording over subsea umbilical cables [42]. While these deployments methods yield data of different quality, the majority of the applications described in this paper can be successful, at least to some extent, with any type of DAS deployment.

A virtual DAS receiver is a single-axis measurement sensor, which is sensitive to strain (or strain-rate) along the direction of the fiber. As a result, DAS’s sensitivity varies as a function of the recorded seismic mode, subsurface properties, and the source location. Traditional downhole receivers, on the contrary, usually have three orthogonal and fixed axes of measurements. This often enables estimating the incidence angle of seismic events using polarization analysis [43,44], which is not possible with DAS measurements. In other words, events recorded with DAS will inherently suffer from cylindrical uncertainty around the fiber. There have been theoretical and practical developments of helical fibers [45,46,47], in which the optical cables are wrapped around a cylinder with specific geometric properties. As a result, the DAS measurement is effectively conducted over angles with different roll, pitch, and yaw. While this installation type can make DAS sensitivity as a function of the incidence angle more uniform, it still does not allow for polarization analysis or resolves the aforementioned cylindrical symmetry. In general, the instrument response of DAS is broader than that of traditional receivers. However, it requires careful treatment and calibration as the fiber properties, casing, and installation method can influence it [31,48,49,50].

Traditional sensors offer a local, or point, measurement. DAS, on the contrary, measures strain over a gauge length [51]. While this characteristic is easy to model and is often harmless, it can prove detrimental in high-resolution applications. Its effect can be modeled as that of a moving average, usually calculated over a boxcar or Gaussian function [52]. Therefore, its effect on the signal will be a function of the seismic wavelength. The gauge length in modern systems is commonly set around 10 m [48], and therefore wavelengths longer than 20 m are relatively unaltered. In most seismological applications, wavelengths of interest are longer. However, when we record shorter wavelengths, the phase and amplitude of recorded signals strongly vary. While the effect of the gauge length is understood and can be modeled, recovering the original signal is difficult due to low SNR. Modern interrogators can have shorter gauge lengths at the expense of lower SNR, which by itself keeps improving with time [9].

DAS-based studies also suffer from uncertainty in mapping recording points to their spatial locations. The distance of each virtual receiver from the laser interrogator is known with high accuracy, but their actual physical locations need to be inferred from the well trajectory and other auxiliary measurements [13]. Even in simple acquisitions within vertical boreholes, there is usually a 1–2% discrepancy between the measured fiber length and the depth of the well [53]. Several approaches have been developed to mitigate this issue [54,55], but in practice, scaling is often based on linear interpolation between a few points.

## 2. Vertical Seismic Profiling

**Figure 2:**
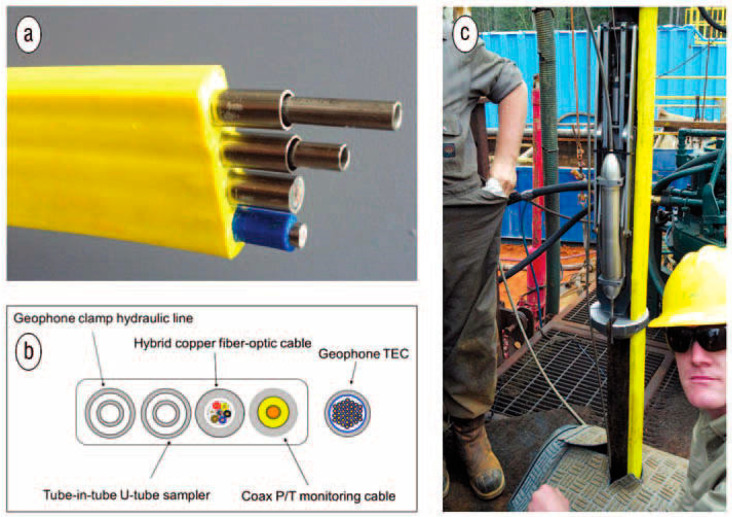
Colocated fiber and downhole geophone installation in early experimental deployment. (**a**) Image of flatpack deployment including the fiber and (**b**) its schematic representation. (**c**) Flat and borehole geophone clamped on the tubing. Figure and caption from Daley et al. (2013), The Leading Edge.

Among the first field examples of DAS was its deployment in hydrocarbon exploration wells for VSP surveys [29,56]. Figure 2 illustrates an early DAS-VSP deployment in parallel with conventional sensors. In traditional VSP surveys, downhole receivers record seismic sources actively generated at the surface [4]. While the term implies usage in vertical boreholes, it nowadays refers to any downhole acquisition. There are multiple possible source configurations for VSP surveys: (1) single source, close to the wellhead (zero-offset VSP) (2) single source, at a fixed distance from the wellhead (offset VSP) (3) multiple sources, with radially increasing distance from the wellhead (walkaway VSP). They can also be conducted along different azimuths, in which case they are referred to as 3D walkaway VSP. (4) for deviated wells, a survey in which there is a source directly above each receiver (walk-above VSP). There are also applications in which VSP records drill-bit noise [57,58], also called VSP-WD (VSP While Drilling). VSP surveys are sometimes repeated at intervals of several years to detect minute changes in the subsurface [59].

VSP surveys serve several purposes. They allow for a high-resolution, albeit local, estimation of the subsurface properties in the proximity of the array. By using zero-offset sources, the recorded seismic traces can be analyzed using a 1-D propagation approximation. By following the arrival times of the downgoing energy, one can accurately estimate the seismic velocity along the vertical direction [60]. While seismic velocities can also be obtained by sonic logging, VSP is conducted with seismic frequencies and thus does not require calibration when used in conjunction with other seismic data [61]. For example, in the presence of subsurface anisotropy, it directly provides effective velocity information that can be used to map reflections to their correct depth. Seismic attenuation [62] can also be directly derived. In VSP surveys with multicomponent receivers, anisotropy parameters can be measured given an adequate source geometry [63,64]. Local, high-resolution estimation of subsurface properties is useful for validating and updating larger-scale models derived from surface seismic surveys. In addition, VSP records can be used to image the subsurface. For that purpose, downgoing energy is filtered out. The upgoing energy, which has been reflected from subsurface discontinuities, can be used for imaging in the vicinity of the array [65]. If this imaging is performed only at the well location, thus in a 1-D fashion, it is referred to as a corridor stack. The obtained image quality depends on the surface source distribution and density and generally decreases with distance from the VSP location. Nonetheless, it usually has a higher resolution than surface seismic surveys, as the seismic energy propagates for shorter distances and thus maintains a higher frequency content. VSP has been especially useful in imaging salt bodies, which are notoriously challenging for surface acquisitions [66,67,68]. Salt-proximity VSP, in which the distance to a salt flank is estimated, is one of its most common applications.

### 2.1. Data Quality and Velocity Estimation

**Figure 3:**
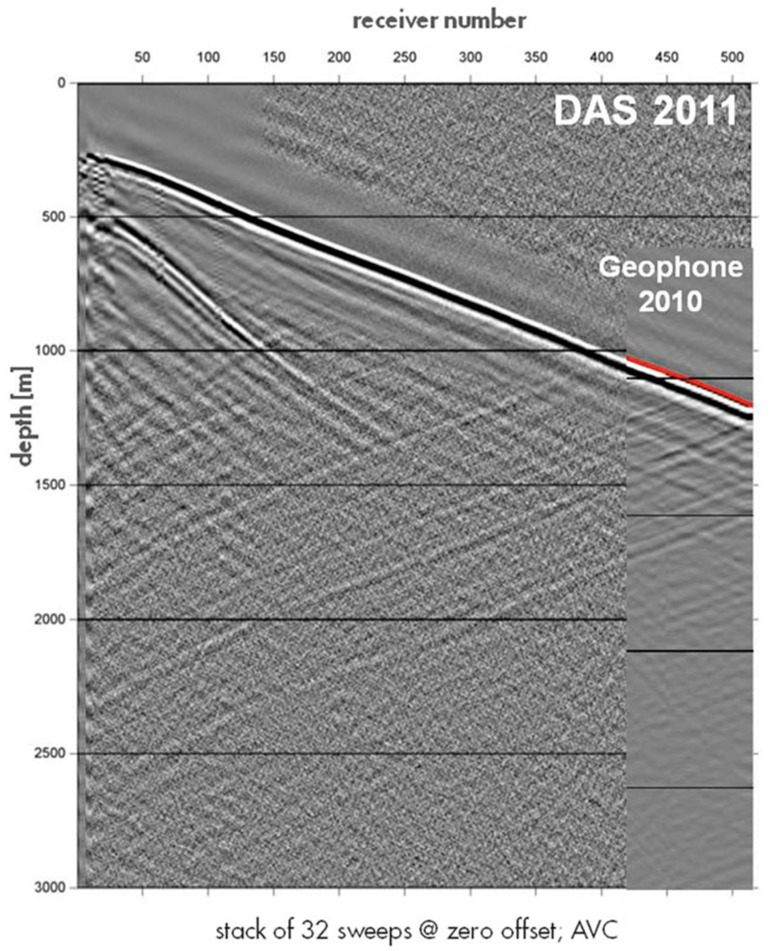
One of the first examples of DAS-VSP records, with data acquired at Pinedale in 2011. Despite a large number of stacked sources, DAS data are noisy. However, downgoing and upgoing P-wave energy is visible, demonstrating the potential of DAS for VSP studies. For shallow DAS locations, the direct S-wave is also visible. DAS measurements are in good agreement, kinematically, with downhole geophones deployed in the same well. Figure from Mateeva et al. (2013), The Leading Edge.

**Figure 4:**
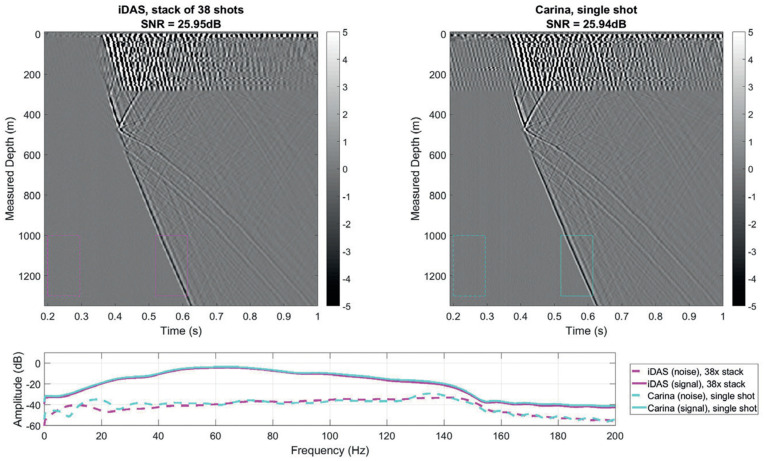
DAS data quality acquired with a modern interrogator, using regular and engineered fiber. For a regular fiber (**left**), a stack of 38 shots is required to obtain the SNR of a single shot recorded with an engineered signal (**right**). Recorded amplitude as a function of frequency, using both fibers, is at the bottom. Figure from Naldrett et al. (2020), First Break.

DAS-based VSP acquisitions are approximately a decade old. Initially, fibers were primarily deployed behind the casing. One of the first published examples is from 2011, in a deep onshore well in Pinedale, Canada [56]. A large variety of deployments in different environments, such as hydrocarbon reservoirs, glaciers, geothermal fields, and carbon sequestration sites, have been reported since then [34,36,69,70,71]. Figure 3 shows a DAS-VSP record from 2011 and its excellent match to a downhole geophone survey conducted a year earlier. While DAS records at the time were significantly noisier, there is an excellent kinematic match of the different seismic phases, both downgoing and upgoing. Analysis of similar data at the Quest CCS project has been shown to yield an excellent match with seismic velocities estimated using both downhole geophones and sonic logs [72]. Over time, the quality of DAS recording systems has constantly been improving. Figure 3 shows data from modern DAS-VSP acquisitions, which is of much higher SNR. It also shows the benefits of using engineered fibers: the same SNR can be obtained by recording a single source with an engineered fiber or summing 38 applications of the same source with a standard fiber. Engineered fibers [41] are purposedly designed to have stronger light backscatter, thus improving recorded signals at the interrogator. They have been shown to potentially improve signals by 10–20 dB [34], as illustrated in Figure 4. 

Recent studies show high-quality DAS data acquired in wells that are not permanently equipped with an optical fiber. Through tubing or wireline deployments, any well can be transformed into a DAS array. There is substantial noise associated with such deployments in vertical wells, mostly due to poor coupling of the fiber to the borehole and ringing, but various approaches have been developed for its mitigation [73,74,75]. In deviated wells, coupling through gravitation is significantly better. A recent study [70] shows that deploying fibers through a wireline in a vertical borehole can yield high-quality data given that the fiber tension is chosen to maximize SNR. Even in the challenging case of a fiber installed inside a flowing tubing [39,76], adequate data may be obtained after adequate processing.

### 2.2. Structural Imaging

**Figure 5:**
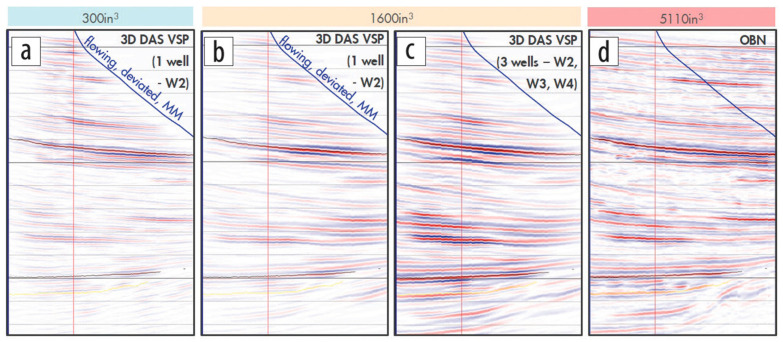
DAS-VSP conducted in flowing wells, with image quality as a function of acquisition efforts (**a**) Image obtained with a small seismic source recorded by a single DAS well. In (**b**), a larger source is deployed, improving the illumination of targets deep below the well. When the same source is recorded by three different DAS wells (**c**), the image extent and continuity are much improved. (**d**) a comparison to the costly alternative—an OBN survey with a very large seismic source. Figure from Kiyaschenko et al. (2020), The Leading Edge.

The quality of recorded DAS data allows for active seismic imaging in the vicinity of the reservoir. There have been many examples, in- and off-shore, of successful imaging [37,77,78,79,80]. Figure 5 shows imaging results obtained with different DAS configurations compared to an ocean bottom node (OBN) survey. OBN is a costly survey in which seismic receivers are laid on the seabed floor, eventually yielding state-of-the-art seismic images. DAS-based images obtained from multiple wells are of similar quality to the OBN results, despite DAS recording being conducted in flowing wells, which are noisier [81]. A single DAS well, nonetheless, has worse illumination of subsurface structures, especially far away from the fiber.

**Figure 6:**
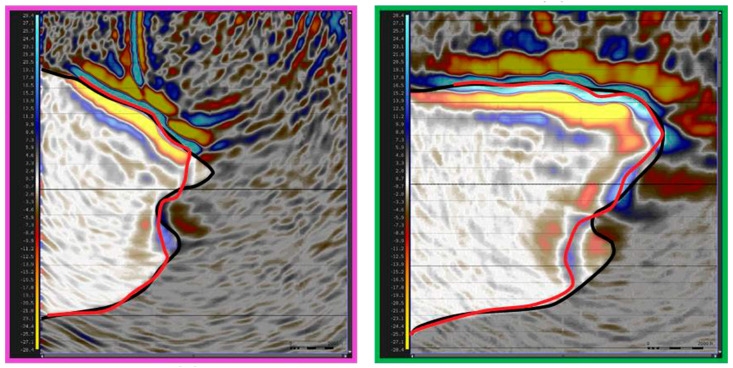
Legacy and DAS-VSP salt imaging, taken along two perpendicular lines. In both lines, the legacy salt interpretation (black) agrees with the DAS-based image (red) for the top of the salt. However, there are significant differences in the salt flank. Figure from Duan et al. (2020), SEG Annual Meeting Expanded Abstracts.

DAS has also been successfully used for the imaging of salt bodies. In a recent study [82], it has been shown to outperform legacy surface seismic data. Figure 6 shows an inline and crossline view of a salt body imaged with DAS-VSP and its comparison to a legacy seismic image. While the top of the salt body is similar, the flanks are better delineated with DAS, and the structure is clearer than in the legacy image. Imaging of a polymetallic mine has also been conducted with DAS-VSP [83].

### 2.3. Time-Lapse Analysis

If the DAS fiber is installed behind the casing, repeating active surveys is a relatively straightforward task as the receivers’ locations and response functions are fixed. The challenge of having perfectly repetitive sources still persists, but it is the case for any time-lapse (also known as 4-D) seismic survey. Time-lapse DAS VSP has been shown to reach high repeatability levels even for semipermanent tubing deployments. In the Gulf of Mexico, deepwater DAS measurements reached a 7% discrepancy between surveys, measured through the normalized root mean square (NRMS) method [84], a figure which is considered excellent [53]. Therefore, 4-D surveys can also be reliably conducted in wells that are not instrumented with DAS. With the installation of permanent sources [85], surveys can be repeated easily.

**Figure 7:**
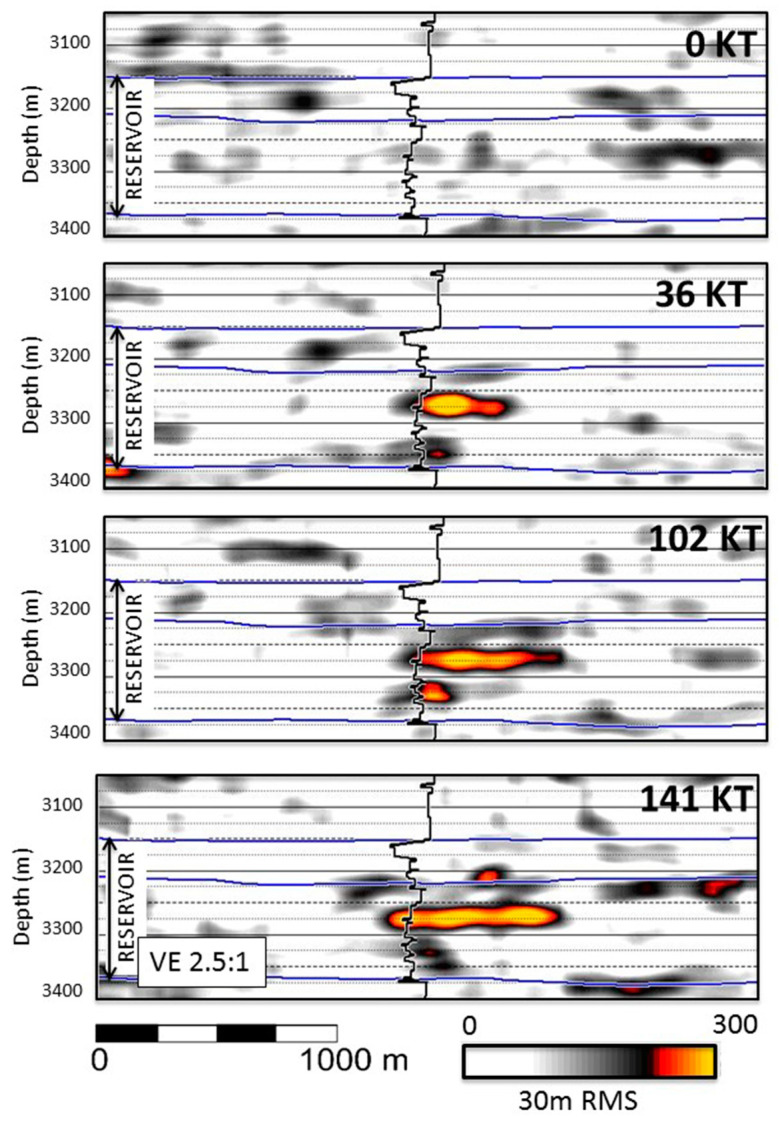
Time-lapse DAS-VSP imaging tracking a CO_2_ plume. As the volume of injected CO_2_ increases, the amplitude change relative to the baseline image increases. Figure from While et al. (2020), SEG Annual Meeting Expanded Abstracts.

Time-lapse DAS VSP has been used to track CO_2_ in the Aquistore project in Canada [86,87] and South West Hub in Australia [88]. The reflectivity of the target reservoir varies due to the injection of CO_2_, and these changes can be detected by a time-lapse of DAS records. Figure 7 shows the imprint of different amounts of injected CO_2_ on the residual DAS-VSP image in the Aquistore project. At low amounts, changes are random and are due to imperfect repeatability. However, after more CO_2_ has been injected, the change is clearly visible. These changes have also been observed with a surface geophone array.

DAS-VSP can also be used to track subsurface changes in hydrocarbon reservoirs [36,81]. Seismic properties may vary due to the stimulation of unconventional reservoirs [89,90,91] and production, primarily important in conventional ones [36]. For example, [91,92] show how 4-D DAS-VSP acquired in a deviated well can delineate seismic signatures related to fractures opening during stimulation. The kinematic and dynamic properties of these signals can be used to infer the vertical fracture geometry and their temporal behavior.

### 2.4. VSP Analysis with Passive Recording

**Figure 8:**
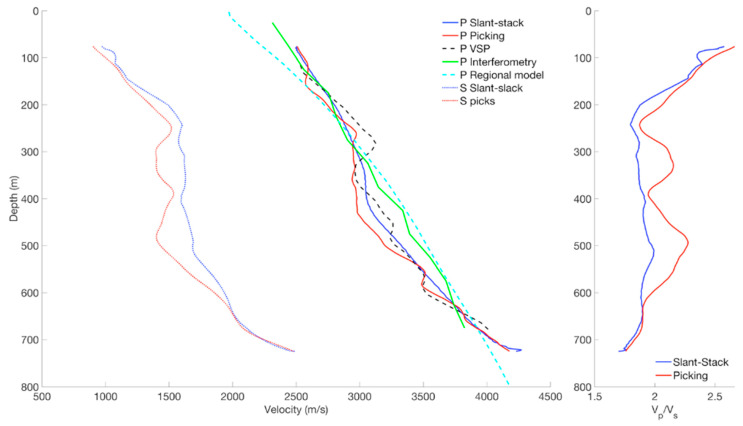
Seismic velocities estimated in a DAS-VSP well. The result of a zero-offset VSP survey with geophones (dashed black line) agrees very well with velocities derived using a recorded earthquake (solid blue and red lines). Earthquake analysis also yields a shear-wave velocity profile (dotted blue and red) which cannot be obtained from the VSP survey. Ambient seismic signals can be used to recover P-wave velocity (green) with low-frequency resolution. Figure from Lellouch et al. (2019), Journal of Geophysical Research: Solid Earth.

Subsurface properties along a DAS-VSP array can also be obtained from passive recordings. Earthquakes, for example, can generate seismic events that propagate through the DAS-VSP array [10]. By following their time of arrival, one can estimate the seismic velocities of the subsurface. Figure 8 shows seismic velocity models derived from an earthquake recorded in a DAS-VSP array. It has excellent agreement with the results of a geophone-based zero-offset VSP survey. In addition, it can retrieve the shear wave velocity, which is impossible in zero-offset VSP. The same approach can also be conducted using the ambient seismic field instead of a particular earthquake, in this case utilizing one-day intervals. This data allows estimating the P-wave velocity, albeit with lower resolution. As ambient noise analysis can be continuously conducted, it is highly beneficial for 4-D studies.

Other subsurface properties can also be analyzed using earthquake records in DAS-VSP. By measuring DAS amplitudes and applying energy conservation principles, one can infer a velocity- and density-dependent quantity along the well [93]. Therefore, a joint analysis of these two methods can yield independent density values in addition to the velocity. Similarly to traditional VSP analysis, attenuation properties could also be derived.

## 3. Seismic Monitoring

### 3.1. Microseismic

Hydraulic stimulation, or hydrofracking, is the underlying technology behind unconventional hydrocarbon recovery. Fractures in the reservoir are created or activated through the injection of pressurized fluid [94]. Proppant is added to the hydrofracking fluid to keep the fractures open once the injection stops. At this point, hydrocarbons will flow to the well through the network of open fractures. Microseismic monitoring is the key technology in imaging the fracture network induced by hydraulic stimulation [5]. As fractures open, they release faint seismic energy that can be recorded with adequate arrays. With downhole receivers located close to the fractures, seismic events with magnitudes as low as M = −3 can be reliably detected. They are subsequently located and, the geometry of the acquisition array permitting, their magnitude and focal mechanism are resolved, indicating the extent and orientation of the faulting plane [95]. Receivers can be deployed at the surface, but because the seismic waves travel through a considerable distance, and often through complex near-surface layers, their usefulness for all stages of microseismic analysis is often limited [96,97,98].

The deployment of downhole receivers is costly. A dedicated well needs to be drilled, as the sensors cannot be placed in a fluid-flowing well. Electronic components are limited in the temperature and pressure range at which they can operate. To allow for a reasonable array coverage, the distance between receivers is on the order of tens of meters, and short wavelengths may be spatially aliased. Therefore, at first sight, DAS is a natural alternative for microseismic monitoring, especially if the fibers can be installed behind the casing. As the recording fiber is close to the microseismic events, a higher SNR and resolution can be expected. While several successful case studies, highlighting the potential of DAS-based microseismic, have been published, it is not as routinely conducted as DAS-VSP. The primary limitation is the uniaxial nature of DAS. As a result of the cylindrical symmetry that follows, DAS in a single well cannot, generally speaking, locate microseismic events, and only certain focal planes can be resolved. In addition, individual DAS channels are usually noisier than conventional receivers [30]. Therefore, the most widespread microseismic analysis methods, based on identifying distinct seismic phases at each receiver, underperform when applied to DAS data. Finally, microseismic information is most valuable in near-real-time [99]. The amount of data generated by DAS imposes significant computational difficulties to the timely delivery of results.

**Figure 9:**
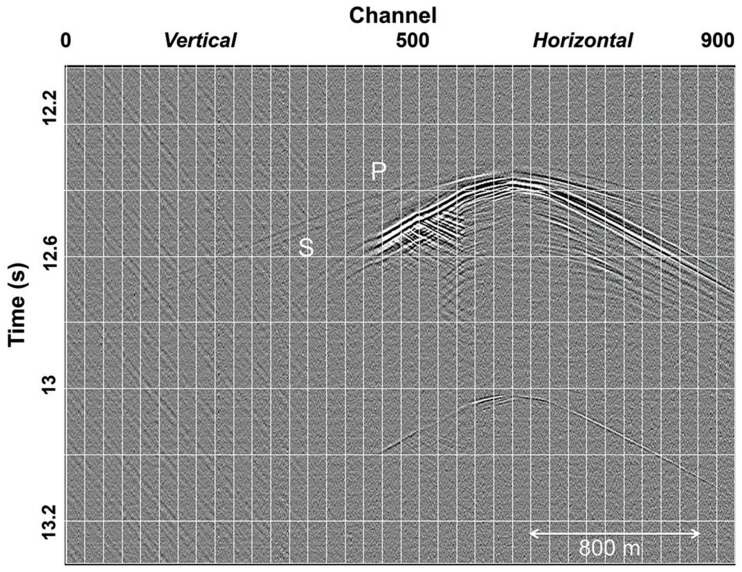
A microseismic event recorded with DAS in a deviated well. Direct P and S arrivals are visible and marked, but there are also reflections and coda events. Recording in the vertical part of the well has much lower SNR, as can also be seen in the later microseismic event that is only visible in the horizontal section. Figure from Karrenbach et al. (2017), The Leading Edge.

The ability to passively record microseismic events with DAS has been recognized and validated using colocated conventional receivers as early as 2013 [100]. As the main application of microseismic monitoring is in unconventional reservoirs, fibers are often deployed in deviated wells that target the reservoir. Figure 9 shows an example of a microseismic event recorded over such a deviated fiber. Early applications of conventional detection algorithms to DAS were relatively unsuccessful. For example, for monitoring hydraulic stimulation [101], 31 DAS events were found compared to 785 with conventional geophones. In [102], the DAS-based detection event count is reported to be 10% of the geophone-based one. Furthermore, [103] shows the DAS can only detect the events of the largest magnitude. Notwithstanding, as illustrated Figure 9, microseismic events visible on DAS displayed interesting waveform characteristics, including mode conversions and reflections/scattering. Despite limitations in early detection capabilities, event location was attempted [102,104]. While the benefits of DAS-based location, mostly in positioning the event along the fiber axis, unfolded, the symmetry problem arising from recording on a single fiber limited the ability to retrieve unambiguous locations.

**Figure 10:**
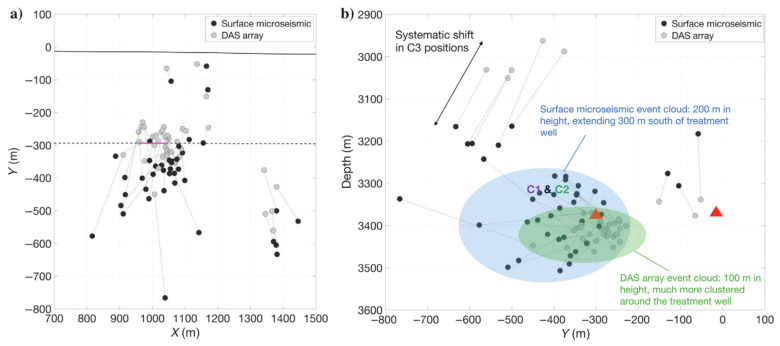
Comparison between event locations derived from two deviated DAS wells (light gray) and surface geophones (black) in map (**a**) and cross-section (**b**) views. DAS locations are generally more clustered around the stimulated well (pink line in **a**). In (**b**), red triangles denote the locations of the wells. Figure from Verdon et al. (2020), Geophysics.

Using a single vertical fiber, [105] showed a beamforming approach that is useful for event detection and event location without azimuthal information. DAS detection capabilities were at about 30% of those of conventional geophones, which shows a significant improvement compared to the application of standard detection methods [106]. In unconventional reservoirs, where horizontal DAS fibers are deployed, [107] show that after trace-by-trace picking, DAS recordings can be used for travel-time minimization based on a known velocity structure. Despite the cylindrical symmetry, several assumptions based on production logs can be made to yield reasonable uncertainty. For certain events, it is possible to detect arrivals in the deviated and vertical parts of the well, in which case many previously degenerate planes can be resolved. Furthermore, [108] uses deviated well recordings to approximately estimate event location without the need for individual channel picking. Instead, they measure several geometrical quantities, evident in the DAS records, and locate events assuming a constant background model. They also observe that downhole DAS outperforms a surface recording array, which is commonly used for microseismic monitoring [97], by close to an order of magnitude in terms of detected events. Figure 10 shows that event locations are more clustered than those obtained with surface geophones. Machine learning approaches have also been attempted and have shown reasonable performance [109,110]. However, they are highly dependent on the size and quality of training datasets, and substantial effort is needed in generating those. If trained neural networks can prove to easily generalize across different sites, such approaches have vast potential for event detection. They simultaneously utilize spatiotemporal patterns across hundreds or thousands of channels and can thus overcome the lower SNR of individual channels.

DAS can also be used for focal mechanism [99,111] and magnitude [14,105] estimation. For both, the directional measurement of DAS poses a significant challenge, as not all components of the seismic wavefield excited by the source can be recorded. Therefore, there are inherent limitations to the focal planes that can be resolved for a given recording geometry. A prominent feature of DAS records is polarity flips in the data. Their position depends on the focal mechanism and can thus be used to estimate it. For magnitude estimation, instrument response is critical [48], but it is not always possible to directly calculate it in downhole deployments. The most straightforward solution is to calibrate the DAS array’s response with a conventional downhole geophone, but it is not always practically possible. Simultaneous DAS recording in multiple wells can significantly alleviate many of these limitations [112].

### 3.2. Earthquake Seismology

In earthquake seismology, events are significantly more distant from the DAS array than for the microseismic case. As a consequence, the focusing power of the array diminishes. In addition, the high spatial density is not always directly useful, as recorded events underwent attenuation and thus have much longer wavelengths, for which the meter-scale resolution of DAS is not necessary. Oftentimes, earthquakes are not the primary target of downhole DAS deployments [80,93]. As a result, they are not necessarily studied for seismological purposes.

Nonetheless, downhole DAS can provide a significantly more sensitive detection array compared to surface deployments. A recent study [14] shows that a vertical downhole DAS array can outperform a surface-based regional seismic network by an order of magnitude. [17] showed that downhole DAS performed similarly to a dense surface array. This benefit arises from the ability to record events closer to their source and, most importantly, before they propagate through the highly dissipative heterogeneous shallow layers of the Earth. In addition, all DAS channels can be simultaneously utilized as a beamforming array, thus overcoming the lower SNR of individual channels.

**Figure 11:**
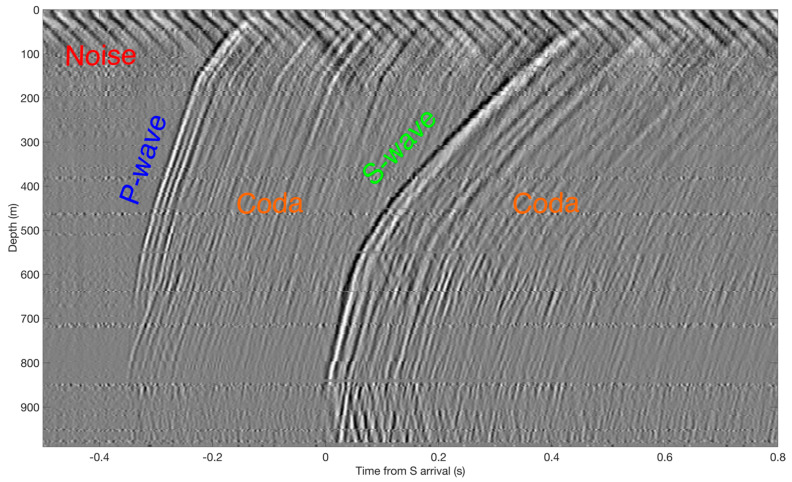
Earthquake recorded by vertical DAS. It shows clear P (annotated in blue) and S arrivals (green), as well as trailing coda events (orange). First arrivals have a refraction-like behavior, as the energy dissipates quickly below a depth of ~840m. High levels of noise (red) in the near-surface are visible, illustrating the benefits of downhole recording. Adapted from Lellouch et al. (2021), Journal of Geophysical Research: Solid Earth.

The rich wavefield recorded by DAS can explain many physical phenomena that are hard to recover using conventional surface arrays. For example, Figure 11 displays clear P- and S- wave arrivals and shows that the event has a refractive behavior. The first arrival of both phases is at a depth of ~800 m and not the bottom of the array. Therefore, the authors determined that this event originates in the sedimentary area and refracts along the top of the bedrock. Such insights cannot be obtained from a surface array. The rich coda energy, visible in the same figure, can be used in studies about earthquake-induced changes to the subsurface [21].

## 4. Reservoir Characterization

### 4.1. Fracture Detection with Low-Frequency DAS

**Figure 12:**
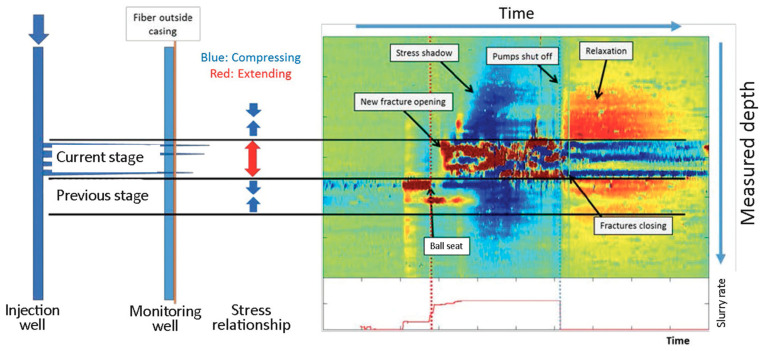
Low-frequency DAS recording of fractures and pressure fronts interacting with a fiber in an offset well. Events of interest are labeled. Figure from Jin and Roy (2017), The Leading Edge.

The ability to deploy DAS fibers in wells located inside unconventional hydrocarbon reservoirs offers unprecedented opportunities in reservoir monitoring [101,104,113]. DAS is a broadband instrument whose response function reaches frequencies lower than 0.001 Hz [48]. Low-frequency (<0.05 Hz) DAS records have been used to directly characterize events when fracture and pressure fronts hit the well where the fiber is installed due to hydraulic stimulation. A groundbreaking study [114] observed low-frequency DAS signals that could be directly interpreted as fractures opening and closing. In addition, pressure fronts were detected as wider-aperture low-frequency events. Figure 12 shows such a record. Furthermore, [115] illustrates how machine-learning approaches can be used to detect fracture hits in the DAS data. The different interpretation components have been validated by fracture modeling [116,117] and analysis at different sites [41,107,118]. A recent study [119] applied a similar analysis to a multiwell DAS acquisition and could track fracture trajectories and propagation velocity by their intersection with the DAS-equipped wells. Conventional downhole tools are not sensitive in this frequency range. Therefore, DAS offers a truly unique measurement. In addition, its high spatial resolution allows for accurate delineation of multiple fracture systems. After adequate filtering, recorded data volumes for low-frequency DAS can easily be processed in quasi-real-time and efficiently inform decision-makers.

### 4.2. Analysis and Use of Guided Waves

Guided waves have been observed and used for different seismic studies: imaging coal seams [120], studying fault zones [121], crosswell continuity mapping [122], and near-surface property estimation [123]. An area of lower velocity than its surroundings can trap seismic energy, which propagates for long distances with minimal losses. The authors of [124] report DAS recording of guided waves generated from perforation shots with a frequency content of up to 700 Hz, which is approximately an order of magnitude higher than conventional DAS-VSP surveys. The fiber was deployed on a well drilled into an unconventional reservoir, thus allowing for the in-situ recording of guided waves with unprecedented resolution. As guided waves can be generated by microseismic events as well, different applications have been developed following this study.

The authors of [125,126] show how the existence of guided waves in recorded DAS data can be used to infer if their source, a microseismic event, is located inside or outside the reservoir. They find that for events originating inside or close to the reservoir, strong guided waves are generated. Since these guided waves have unique dispersive properties, they are easy to detect automatically. If, on the contrary, events originate far from the reservoir, they generate feeble guided waves. When using surface monitoring arrays, the depth accuracy is very low; guided-wave analysis can cull the events that are likely to influence production.

Thanks to the high resolution of DAS and the proximity to seismic sources in the reservoir, guided waves can be recorded with very high frequencies. Wavelengths as short as several meters can be recorded without aliasing. Therefore, guided waves are an excellent source of information for seismic inversion. Furthermore, [127] uses the dispersive properties of guided waves excited by microseismic events to invert for reservoir properties. Their inversion is for a 1-D layered media, but it includes VTI anisotropy and is validated by vertical logs. Full-waveform approaches are also being developed.

**Figure 13:**
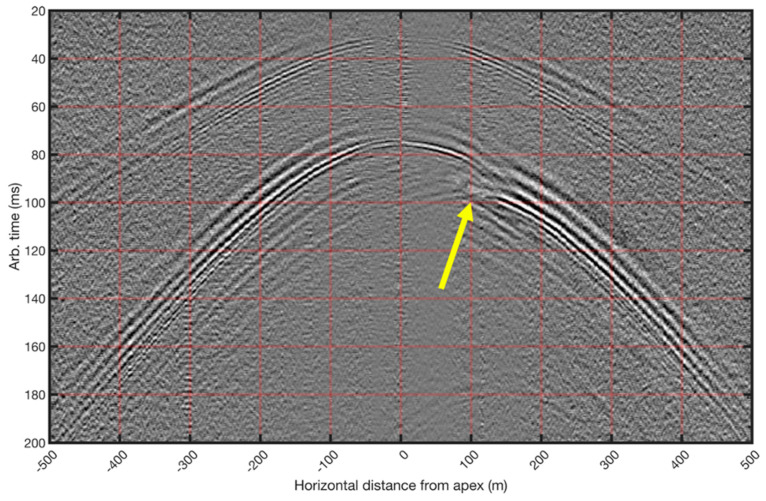
Guided waves and their interaction with fractures. Two sets of arrivals, both dispersive, can be observed. A clear imprint (yellow arrow) from open fractures can be seen on the slower guided S-waves. Figure adapted from Lellouch et al. (2020), Geophysics.

Guided waves can directly illuminate fractures in the reservoir, as illustrated in Figure 13. The authors of [128] show that guided waves generated by perforation shots interact with open fractures, stimulated during preceding stages. Using simple geometrical considerations, they illustrate how perforations from an offset well can be used to estimate the horizontal fracture growth. These estimations are valuable for optimal well spacing and hydrocarbon recovery and can also inform real-time decisions about the stimulation and production.

### 4.3. Tube Wave Analysis

Tube waves have been shown useful to characterize near-borehole conditions, particularly fracture networks [129,130]. As DAS fibers are usually deployed across the entire length of the well, they can follow tube wave behavior in great detail. The authors of [131] show the first example of DAS-based tube wave analysis—by observing perforation-induced tube waves, they follow their decay when crossing previously fractured zones. Synthetic work [132] predicts that pumping noise recorded by DAS can potentially detect near-borehole irregularities as well as fractures opening or closing.

## 5. Conclusions

Downhole DAS offers a high spatiotemporal resolution measurement of the seismic wavefield over a limited area. Its spatial density allows for unaliased recording of short seismic wavelengths and the application of advanced imaging methods. By installing the DAS fiber closer to the events of interest, signal-to-noise is improved, and propagation through the heterogeneous and dissipative shallow subsurface is avoided. This review article summarizes its many active and passive applications: subsurface parameter estimation, structural imaging, time-lapse studies, seismic and microseismic monitoring, and reservoir characterization. As DAS data quality is constantly improving and its cost keeps diminishing, we expect it to become commonplace technology in newly drilled and repurposed boreholes. Its directional nature still imposes substantial limitations on its usefulness, especially for passive applications, but multiple DAS-instrumented wells can alleviate a significant portion of these limitations.

## Data Availability

Not applicable.
